# Functional and phenotypic evaluation of eosinophils from patients with the acute form of paracoccidioidomycosis

**DOI:** 10.1371/journal.pntd.0005601

**Published:** 2017-05-10

**Authors:** Fernanda Gambogi Braga, Luciana Pereira Ruas, Ricardo Mendes Pereira, Xinaida Taligare Lima, Edson Antunes, Ronei Luciano Mamoni, Maria Heloisa Souza Lima Blotta

**Affiliations:** 1 Department of Clinical Pathology, Faculty of Medical Sciences, State University of Campinas, Campinas, São Paulo, Brazil; 2 Department of Pediatrics, Faculty of Medical Sciences, State University of Campinas, Campinas, São Paulo, Brazil; 3 Department of Pharmacology, Faculty of Medical Sciences, State University of Campinas, Campinas, São Paulo, Brazil; Universidad de Antioquia, COLOMBIA

## Abstract

**Background:**

Eosinophilia is a typical finding of the acute/juvenile form of paracoccidioidomycosis (PCM), a systemic mycosis endemic in Latin America. This clinical form is characterized by depressed cellular immune response and production of Th2 cytokines. Moreover, it has been shown that the increased number of eosinophils in peripheral blood of patients returns to normal values after antifungal treatment. However, the role of eosinophils in PCM has never been evaluated. This study aimed to assess the phenotypic and functional characteristics of eosinophils in PCM.

**Methods/Principal findings:**

In 15 patients with the acute form of the disease, we detected expression of MBP, CCL5 (RANTES) and CCL11 (eotaxin) in biopsies of lymph nodes and liver. In addition, there were higher levels of chemokines and granule proteins in the peripheral blood of patients compared to controls. Isolation of eosinophils from blood revealed a higher frequency of CD69^+^ and TLR2^+^ eosinophils in patients compared to controls, and a lower population of CD80^+^ cells. We also evaluated the fungicidal capacity of eosinophils *in vitro*. Our results revealed that eosinophils from PCM patients and controls exhibit similar ability to kill *P*. *brasiliensis* yeast cells, although eosinophils of patients were less responsive to IL-5 stimulation than controls.

**Conclusion/Principal findings:**

In conclusion, we suggest that eosinophils might play a role in the host response to fungi and in the pathophysiology of PCM by inducing an intense and systemic inflammatory response in the initial phase of the infection.

## Author summary

Paracoccidioidomycosis (PCM) is a fungal disease endemic of some Latin America countries. The acute clinical form of the disease, which affects children and young adults, is the most severe form of PCM. It is characterized by a depressed T cell immunity and increased number of blood eosinophils that decreases after antifungal treatment. The role of eosinophils in PCM has never been investigated. We found high levels of eosinophil granules and chemokines in serum of patients. Moreover, patients eosinophils have a higher migratory and adhesion capacity compared to controls. Our results indicate that eosinophils may participate in the early steps of host response to fungi promoting an intense and systemic inflammatory response, which may result in an inefficient immune response against *P*. *brasiliensis* in vivo.

## Introduction

Paracoccidioidomycosis (PCM) is a systemic mycosis caused by dimorphic fungi of the *Paracoccidioides* genus. It is the most prevalent systemic mycosis of Latin America and, in Brazil, it is the leading cause of death among immunocompetent patients [1–4].

PCM is caused by inhalation of environment *Paracoccidioides* conidia. The fungus may remain latent in tissues for years, without any clinical manifestation. Depending on the inoculum or host immune response, the disease may develop into two clinical forms: the acute/subacute form, which affects young adults and children, or the chronic form, which affects older adults [5]. The acute/subacute or juvenile form comprises 10% of all cases. It is the most severe form of PCM, characterized by diffuse lymph node involvement, hepatosplenomegaly and bone marrow dysfunction. It may also affect skin and bones. Young patients of both genders are equally affected [3, 6, 7].

Patients with acute form of PCM have a depressed cellular immune response as evidenced by delayed-type hypersensitivity (DTH) negative tests, deficient lymphocyte proliferation to yeast antigens and the production of Th2 cytokines such as IL-4, IL-5, IL-10 and TGF-β [8]. In addition, these patients produce high levels of IgE and IgG4 antibodies against *P*. *brasiliensis* [9]. Also in this form, eosinophilia had been correlated with negative delayed hypersensitivity skin tests, lower CD4 cells number and high levels of anti-*P*. *brasiliensis* antibodies, in addition to disease activity and severity [10, 11]. This increased number of eosinophils typically returns to normal after antifungal treatment [10, 12–14]. However, little is known about the role of these cells in the pathogenesis of PCM.

The role of eosinophils in health and disease has received more attention in the past decades [15–17]. Eosinophils, commonly correlated with immune responses during allergic and parasitic diseases [18, 19] participate in both innate and adaptive immunity, since it activates and interacts with several immune cells, including dendritic cells and T lymphocytes [20].

Eosinophils are recruited from the circulation to the inflammatory foci in response to various stimuli. Eosinophil degranulation and release of cytotoxic molecules, i.e. MBP, ECP, EPO and EDN, can quickly affect the microenvironment and influence cell recruitment, tissue repair, homeostasis and remodeling, and also promote a direct response against the pathogen [17, 21]. In addition, eosinophils can present antigen to T lymphocytes and, therefore, act as antigen presenting cells (APC) and initiate an immune response to specific antigen [22]. Eosinophils can also act as an effector cell, inducing tissue destruction and dysfunction, as well as promoting exacerbation of the inflammatory response through the release of toxic proteins from their granules, cytokines and lipid mediators [23, 24]. To date, there are no studies evaluating the role of eosinophils in PCM, even though eosinophilia is part of the diagnostic criteria of the acute form [10, 12–14]. The aim of this study was to evaluate the functional capacity of peripheral blood eosinophils in patients with the acute form of PCM.

## Methods

### Ethics statement

All study procedures were performed after informed consent, in accordance with standards established by the Committee of Ethics in Research School of Medical Sciences, UNICAMP (No 449/2008). Written informed consent was obtained from all participating subjects or their parents/guardians (on behalf of child participant).

### Donors

We included 15 patients with the acute/juvenile form of PCM evaluated at a Pediatrics clinics at the University of Campinas, São Paulo, Brazil. The diagnosis was established by direct examination and/or serology (double immunodiffusion test). The control group was composed of 11 healthy individuals with a maximum age of 35 years old. For each patient and control three fecal samples were collected in alternated days and examined microscopically for the presence of intestinal parasites. All experiments were performed with peripheral blood eosinophils obtained at the moment of diagnosis, before the beginning of treatment.

### Fungi

*Paracoccidioides brasiliensis* strain 18 (Pb18) and strain 265 (Pb265) yeast cells were obtained from Microbiology Laboratory (State University of Campinas, Sao Paulo, Brazil) and maintained by weekly subcultivation in Fava-Netto medium at 37°C. After 5 days of culture, yeast cells were washed and suspended in phosphate-buffered saline (PBS, pH 7.2). Fungal suspensions were homogenized with glass beads in a vortex homogenizer in order to obtain individual cells. Yeast viability was determined by Trypan blue exclusion test and we used fungal suspensions containing more than 85% viable cells.

### Immunohistochemical analysis

Biopsies specimens were taken from lymph nodes and liver of acute PCM patients before the beginning of treatment. Samples were fixed in 4% formaldehyde and embedded in paraffin. Immunohistochemical analysis was performed using antibodies for MBP, CCL5 (Santa Cruz, CA, USA) and CCL11 (R&D Systems, MN, USA). A polymer-based method was used (MACH 4 Free biotin-Detection, Biocare Medical, USA), according to the manufacturer´s instructions. Brown staining indicated positive reaction.

### Eosinophil isolation

Eosinophils were isolated using negative selection method, as described elsewhere [25, 26]. Peripheral blood collected in heparin tube from healthy donors and patients with acute PCM before the beginning of treatment. It was then diluted 1:1 with phosphate buffered saline/bovine serum albumin 1% (PBS/BSA 1%) and overlaid onto Percoll gradient (density = 1.088 g/ml), centrifuged at 1000 × *g* for 20 min, 20°C and the pellet containing red cells and granulocytes was collected. Red cells were lysed using lysing buffer (155 mM NH_4_Cl, 10 mM KHCO_3_ 0.1 mM EDTA). For eosinophil purification, granulocytes were incubated with an antibody cocktail of eosinophil isolation kit (MACS, Miltenyi Biotec Inc., Auburn, CA, USA) for 15 minutes. After this period, cells were incubated with anti-CD16 immunomagnetic microbeads for 15 min at 4°C and added to a magnetic column field. The negative cells, eosinophils, were collected. Eosinophils were suspended in Eagle's minimum essential medium (MEM, Sigma Chemical CO., MO, USA), pH 7.2 (> 92% eosinophils). The cell viability (97%) was assessed in the trypan blue dye exclusion test. The purity of eosinophils (CD16^-^CD49d^+^) was verified by FACs and reached > 98%.

### Serum levels of specific granules of eosinophils (MBP, ECP, EDN and EPO) and chemokines (CXCL9/MIG, CXCL10/IP10, CCL5/RANTES and CCL11/eotaxin)

Serum levels of CCL11, CCL5, CXCL9, and CXCL10 of patients and healthy individuals (controls) were quantified by ELISA technique using kits from R&D Systems (Minneapolis, MN, USA). The granule proteins of eosinophils were assessed by commercially available ELISA according to the manufacturer’s instructions. For MBP, EPO and ECP we used USCN Life Science Inc (Wuhan, Hubei, China) and for EDN we used ALPCO immunoassays (Salem, NH, USA).

### Chemotaxis assays

Eosinophil migration was measured using a 96-multiwell ChemoTx 101–5 chamber (NeuroProbe Inc., Cabin John, MD, USA). Eosinophils from patients with acute PCM and healthy volunteers were ressuspended in MEM at a concentration of 3 x 10^6^ cells/mL. First, microplate wells were filled with 29μL of chemotactic agent (CCL5 - 100ng/mL, CCL11 - 100ng/mL or interleukin-5–100 and 50ng/mL) diluted in MEM or only MEM (spontaneous migration). The upper part was separated from the lower chamber by a 5μM polycarbonate membrane. Subsequently, 25μL of eosinophil suspension was added to the polycarbonate membrane. Eosinophils were isolated *ex vivo* or were previously incubated with *P*. *brasiliensis* yeast cells for 4 hours. The chamber was then incubated for 2 h at 37°C in a humid atmosphere with 5% CO_2_. After incubation, the non-migrating cells on the top side of the filter were removed by a tissue and the chamber was centrifuged at 200 *x g* for 5 min at 20°C. The filter was then removed and the number of cells that had migrated into the bottom compartment was determined by measuring residual eosinophil peroxidase (EPO) [27, 28]. Fifty μL of EPO substrate (1 mM H_2_O_2_, 1 mM o-phenylenediamine and 0.1% Triton X-100 in Tris buffer, pH 8.0) were added to each well. After 30 min at room temperature, 25 μL of 4M H_2_SO_4_ were added to each well to stop the reaction and absorbance measured at 490nm in a microplate reader (Bio-Rad Laboratories Inc., Philadelfia, PA, USA). The number of migrated cells was calculated by comparing absorbance of unknown samples to that of the standard curve (eosinophil number versus EPO activity) for cells from controls and PCM patients. The standard curves for cells from control and PCM patients did not differ significantly, indicating that EPO activity can be used as a marker of eosinophil number. We calculated the relative migration by dividing the number of eosinophils that migrated towards the chemoattractants by the number of eosinophils that migrated towards MEM (spontaneous migration). Each experiment was carried out in triplicate.

### Adhesion of eosinophils to human lung endothelial cells (HLEC)

To assess whether eosinophils have the capacity to adhere to endothelial cells we used HLECs. Ninety-six-well plates were prepared by coating individual wells with HLECs in DMEM (100 μl; 2x10^5^cells/mL) for 2 days at 37°C in a humid atmosphere with 5% CO_2_. After this period, fresh DMEM-FBS was added and part of the cells were stimulated with TNF-α (10ng/mL) [29, 30] for 4h. The plates were washed twice again with DMEM-FBS. Eosinophils of patients and controls were incubated for 30 minutes with CCL5 (100ng/ml), CCL11 (100ng/ml) or IL-5 (100ng/mL), as previously documented [23] and then added to HLECs, previously stimulated or not with TNF-α. After 1h incubation with HLECs, non-adhered cells were removed and the remaining cells were washed twice with DMEM-FBS. At the end of the washings, the wells were filled with 50 μL of MEM. Eosinophil adhesion was calculated by measuring residual eosinophil peroxidase (EPO) activity of adherent cells, as previously described [27, 28].

### Expression of T-cell activation markers and PRRs in eosinophils

Characterization of T cells activation markers and PRRs on the surface of eosinophils was performed using flow cytometry. Eosinophils were purified from the peripheral blood of patients with the acute/juvenile form of PCM and controls as described (Eosinophil isolation). Alternatively, some cells were incubated for 4 hours in the presence of *P*. *brasiliensis* yeast after purification. For the flow cytometry assay, eosinophils were incubated with 200μL human AB serum for 10 minutes at 4^°^C. Cells were washed with 1 mL of dilution buffer [PBS-BSA (0.1%), NaN_3_ (0.2 mM)], the supernatant was discarded and the precipitate suspended in dilution buffer. After this procedure, 20μL of the cell suspension were distributed in 96-well plates with U bottom containing the following antibodies: anti-CD16 (FITC), anti-TLR2 (FITC), anti-CD86 (FITC), anti-TLR4 (PE), anti-HLA-DR (PE), anti-CD80 (PE), anti-CD69 (PE-Cy5) e anti-CD49d (PE-Cy5) and isotype controls, diluted in dilution buffer. After 20 minutes incubation at 4^°^C, in the dark, cells were washed with 120μL of dilution buffer. The supernatant was discarded and the cells were suspended in 200μL of 2% formaldehyde. After the transfer to appropriate tubes, the reading was held in flow cytometer (FACScalibur, Becton & Dickson, USA). For each sample, a minimum of 25,000 events were acquired. The results (relative cell percentage for each parameter in the isolated population of eosinophils) were analyzed using FSC Express (v.3, De Novo Software, USA).

### Fungicidal activity of eosinophils against *P*. *brasiliensis*

The fungicidal activity of eosinophils directly against *P*. *brasiliensis* yeast cells was evaluated by incubating eosinophils with *P*. *brasiliensis* in a 1:100 fungus/eosinophils ratio, in a U bottom 96-well plate, for 4h at 37°C. After this period, 100μL of this culture (diluted or not) was plated on brain-heart infusion agar (BHI, Difco Laboratories, MI, USA), which was supplemented with 4% horse serum and 5% *P*. *brasiliensis* isolate 192 culture filtrate. In some cultures we added yeast cells and human rIL-5 (25ng/mL) (Peprotech, NJ, USA). Plates were incubated at 37°C and the number of colony forming units (CFU) were determined from the fifth to the thirtieth day of culture and then corrected by the dilution factor and expressed in number of CFUs/mL.

### Statistical analysis

GraphPad Prism software (v.5, San Diego, CA, USA) was used for statistical analysis. After analyzing data for normality, results were presented as mean ± SEM or median, accordingly. Student's t-test or Mann Whitney U test was used to compare parameters between patients and controls, as appropriate. One way repeated measures ANOVA, followed by Tukey post test, was used to compare different conditions in the same experiment. A P value < 0.05 was considered statistically significant.

## Results

### Patients characteristics

Table 1 shows clinical characteristics of the 15 patients included in the study at the moment of diagnosis, before the beginning of treatment. Patients' ages ranged from 5 to 14 years-old (mean: 9.57 years-old). We decided to include a 32-years-old female patient (patient 15) because she presented clinically with the acute form of PCM, including eosinophilia and lymph node involvement. In agreement with previous data on the acute/juvenile form of PCM, there was no significant difference between the proportion of male and female patients [1, 31]. As expected, lymph nodes and liver were commonly affected [5].

**Table 1:** Clinical characteristics of patients.

Patients	Sex	Age	anti-*P*.*brasiliensis* (titer)	Eosinophils number x 10^3^/mm^3^ (%) *	CRP (mg/L)*	Affected organs
P1	M	9	1/64	2.4 (5)	131.0	Lymph nodes, skin, liver
P2	F	14	1/32	1.40 (19.5)	29.4	Lymph nodes, bones
P3	M	12	1/32	5 (35.4)	90.9	Lymph nodes, skin, liver
P4	F	7	1/32	1.50 (8)	146.0	Lymph nodes, bones
P5	M	13	1/32	9.68 (34.8)	155.0	Lymph nodes, skin, liver
P6	M	5	1/16	1.88 (8)	93.2	Lymph nodes, liver
P7	F	8	1/8	2.36 (18.2)	126.0	Lymph nodes, skin, liver
P8	M	7	1/32	0.29 (3.3)	89.9	Lymph nodes
P9	M	8	1/32	2.54 (17)	154.0	Lymph nodes
P10	M	11	NR	2.32 (16)	62.4	Lymph nodes
P11	F	10	1/16	0.35 (4.7)	139.0	Lymph nodes, skin
P12	F	11	NR	0.65 (7)	175.0	Lymph nodes
P13	F	9	1/2	12.93 (61)	15.5	Lymph nodes, skin
P14	F	10	1/32	6.63 (28.6)	98.5	Lymph nodes
P15	F	32	1/4	0.82 (7.9)	3.8	Lymph nodes

*Reference values: Eosinophil number: 0.04–0.5 x 10^3^; CRP < 5mg/L; NR: non- reagent. # Anti-gp43 antibodies title (ELISA) for patients P10 and P12 were 1/51,200 and 1/12,800, respectively.

The absolute number of eosinophils in peripheral blood was found to be increased in most patients, with exception of two. Cell counts ranged from 0.29 to 12.93 x 10^3^/mm^3^. The anti-*P*.*brasiliensis* antibody was detected in the serum (immunodiffusion test—ID) of most patients (Table 1). For those patients presenting with a negative immunodiffusion (P10 and P12) we performed ELISA tests using *P*. *brasiliensis* gp43 as antigen and detected antibodies at 1/51.200 and 1/12.800 titers, respectively (Table 1). C-reactive protein levels were elevated in most patients, ranging from 3.8 to 175.0 mg/L.

All patients tested negative for intestinal parasitosis and did not report any allergies.

### Lymph nodes and liver lesions of PCM patients express MBP, CCL5 and CCL11

**Fig 1:**
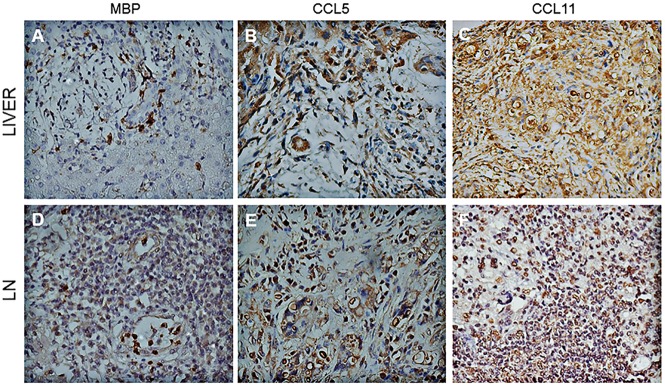
Immunohistochemistry detection of chemokines and granule proteins in liver and lymph nodes from PCM patients. MBP (A,D), CCL5 (B,E) and CCL11 (C,F) were stained in cells distributed in inflammatory infiltrates of lymph nodes (LN) and liver from PCM patients (Original magnification x 400).

Liver and lymph nodes biopsies of patients with the acute/juvenile form of PCM were evaluated by immunohistochemistry in order to detect MBP, CCL5 and CCL11. Fig 1A and 1D show cells with morphology of eosinophils expressing MBP in the inflammatory infiltrate around fungal cells in the liver and lymph nodes, respectively.

CCL5 was detected in areas of hepatic parenchyma, as well as in giant cells (Fig 1B). In lymph nodes, CCL5 was mainly detected in giant cells, around *P*. *brasiliensis* yeast cells. CCL11-stained cells were diffusely demonstrated in the hepatic parenchyma (C) and lymph nodes (F). Altogether, the examined biopsies showed rich eosinophils infiltrates in close contact with fungal cells and MBP, an eosinophilic granule protein. In accordance, CCL5 and CCL11, chemokines involved in eosinophils recruitment, were also detected in *P*. *brasiliensis* infected tissues.

#### PCM patients present higher serum levels of eosinophilic granules and chemokines than healthy controls

**Fig 2:**
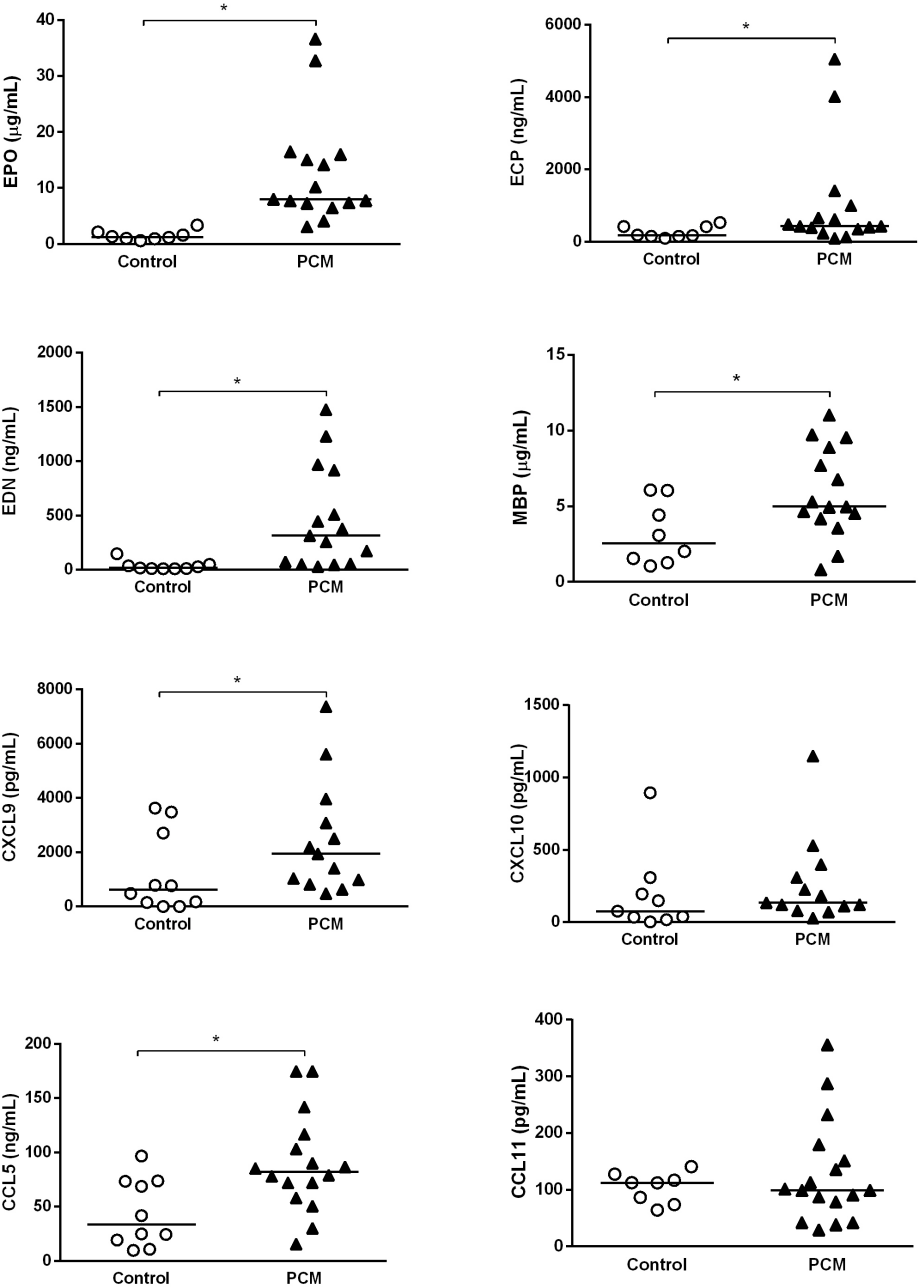
Serum concentration of EPO, ECP, EDN, MBP, CXCL9, CXCL10, CCL5 and CCL11 in patients (n = 13–16) with acute form of PCM and controls (n = 8–10). Results are expressed as median. *Mann Whitney test* * p <0.05.

After demonstrating MBP, CCL5 and CCL11 in tissues of affected organs and presence of eosinophilia, we next evaluated levels of these and other eosinophilic granules and chemokines in sera. Compared with healthy controls, patients with PCM exhibit higher levels of chemokines (CCL5 and CXCL9/MIG) and granules cytotoxic proteins (MBP, ECP, EPO and EDN). There were no significant differences in CCL11 and CXCL10/IP10 levels between patients and controls (Fig 2).

#### Peripheral blood eosinophils of PCM patients present higher migratory capacity to CCL11 than eosinophils of healthy controls

**Fig 3:**
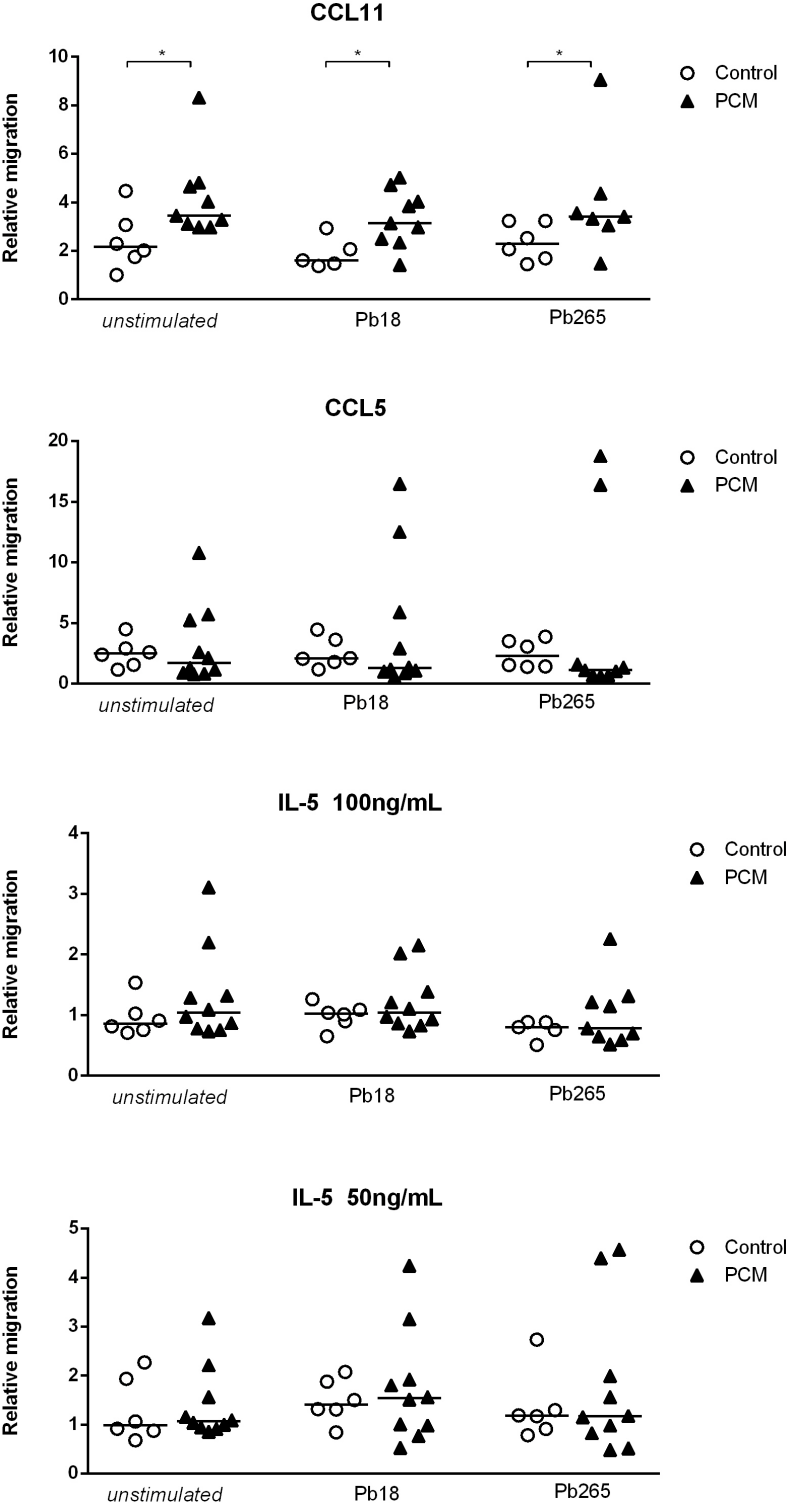
Relative migration of eosinophils of patients with PCM (n = 7–10, black triangle) and controls (n = 5–8, open circles) to CCL11 (100ng/mL), CCL5 (100ng/mL) and interleukin-5 (100 and 50 ng/ml). Eosinophils were maintained in the presence or not of *P*. *brasiliensis* yeasts (Pb18 or Pb265) for 4 hours, as indicated. Results are expressed as median. *Mann Whitney test* * p <0.05.

The traffic of eosinophils to the inflammatory sites involves a series of cytokines, adhesion molecules and chemokines such as CCL5, CCL11 and IL-5 [32, 33]. We evaluated the migratory capacity of eosinophils of patients and controls, in response to stimulation with cytokines, in the presence or not of *P*. *brasiliensis* yeast cells (Fig 3). Eosinophils from PCM patients exhibited higher migratory activity in response to CCL11 when compared with eosinophils from controls. On the other hand, stimulation with CCL5 or IL-5 (50 and 100 ng/mL) did not result in migratory activity. Addition of *P*. *brasiliensis* yeast cells (Pb18 and Pb265) did not interfere with the eosinophils migratory activity in any condition (Fig 3).

### Peripheral blood eosinophils from PCM patients present higher adhesion capacity to human lung endothelial cells (HLECs) than eosinophils from healthy controls

Regulation of expression of adhesion molecules is crucial in controlling inflammation, and the adhesion of leukocytes to endothelial cells is the first step in the recruitment of these cells to the inflammatory sites. Eosinophils express adhesion molecules such LFA-1 and Mac-1, which interact with endothelial cell through ICAM-1 [34].

**Fig 4:**
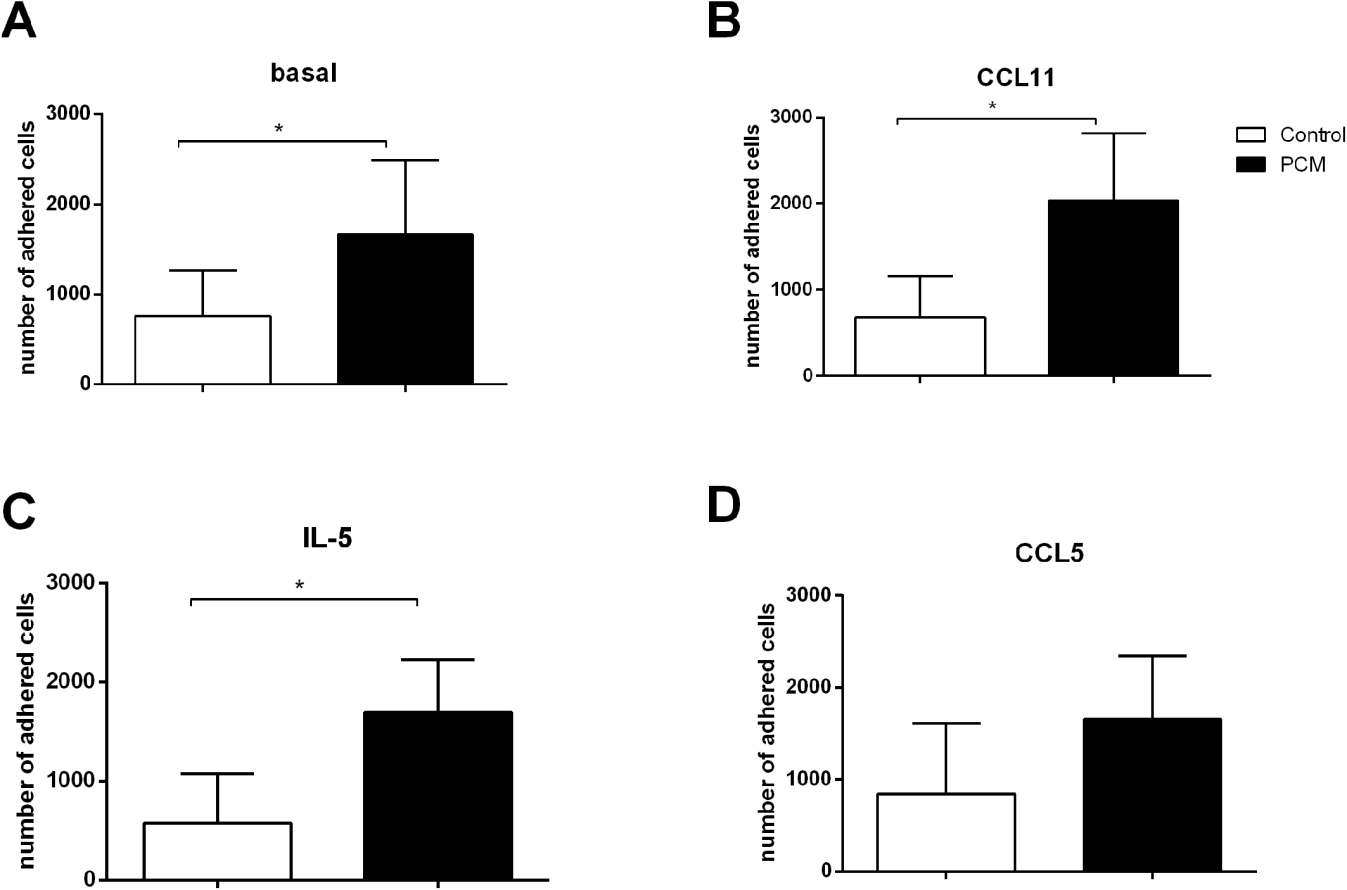
Number of eosinophils from patients with PCM (n = 5, black bars) and healthy controls (n = 6, white bars) adhering to human lung endothelial cells (HLECs). Eosinophils were pre incubated for 30 min with (A) culture medium (basal), (B) CCL11 (100ng/ml), (C) IL-5 (100ng/ml) or (D) CCL5 (100ng/ml) prior to incubation with HLECs for 1 hour. Results are expressed as mean ± SEM. *Student t test*: * p<0.05.

In addition to demonstrating an increased production of chemokines and granule proteins in the serum of PCM patients, as well as higher *in vitro* migratory capacity, in response to CCL11, we evaluated the adhesion of eosinophils to lung endothelial cells (HLECs). Under baseline conditions, eosinophils of patients had a higher adhesive capacity than the ones from controls (Fig 4A). We also stimulated eosinophils with CCL5, CCL11 and IL-5 to evaluate the effect of these inflammatory mediators in the adhesion of eosinophils to HLEC. The stimulation with CCL11 and IL-5 did not change the adhesion capacity of eosinophils (from both PCM and controls) (Fig 4B and 4C). The stimulation of eosinophils with CCL5 promoted a slight increase in the adhesion of control eosinophils to HLECs, but not PCM eosinophils (Fig 4D).

### Peripheral blood eosinophils from PCM patients express more CD69 and TLR2, and less CD80 than eosinophils from healthy controls

**Fig 5:**
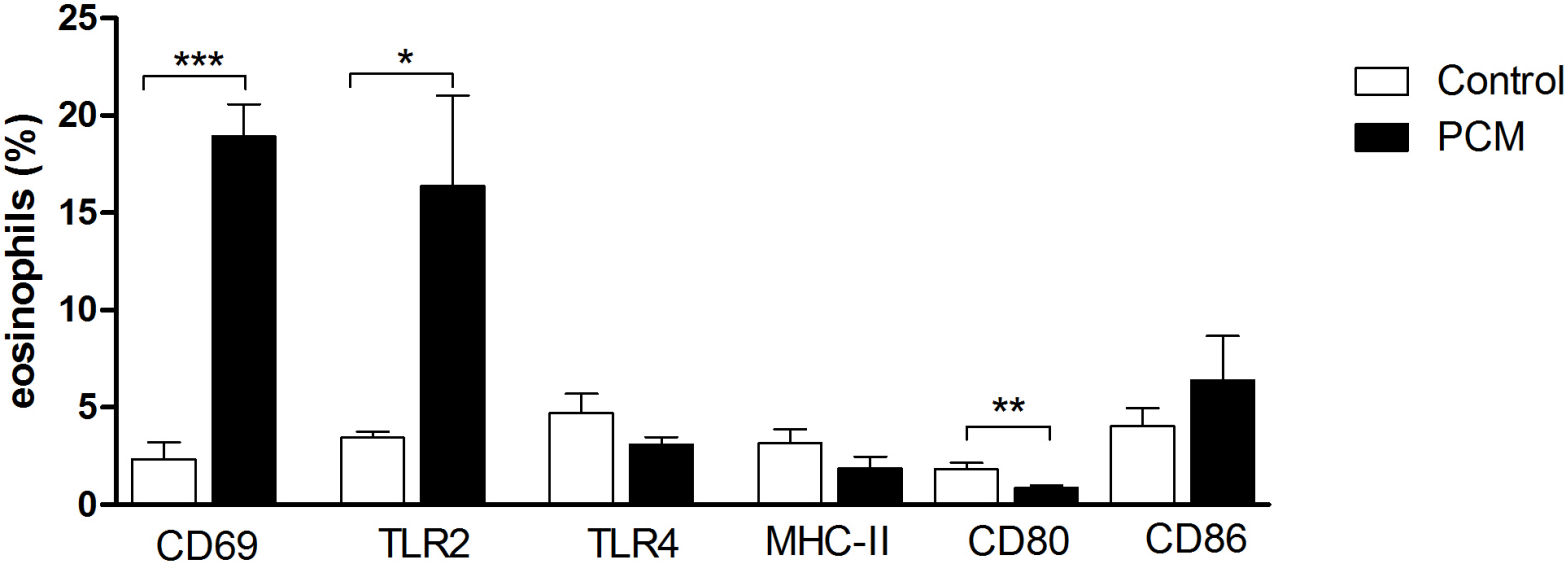
Frequency of eosinophils expressing activation markers, PRRs and T-cell activation molecules in PCM patients (n = 12, black bars) and healthy controls (n = 8, white bars). Peripheral blood eosinophils were purified and incubated with antibodies against human CD69, TLR2, TLR4, MHC-II, CD80 and CD86 and the frequency of each population was evaluated by FACs. Results are expressed as mean ± SEM. *Student t test*: * p <0.05, ** p <0.01, *** p <0.001, compared to control cells.

Given that we found a higher migratory and adhesion capacity in eosinophils obtained from PCM patients, in addition to increased serum chemokines and granule proteins, we evaluated whether the disease would lead to an increment in activated eosinophils in peripheral blood from patients. We then analyzed the frequency of eosinophils positive for the activation marker CD69 in the peripheral blood from PCM patients and controls. We demonstrated a much higher frequency of CD69^+^ eosinophils in PCM patients than in controls (Fig 5).

In the analysis of the frequencies of TLR2^+^ and TLR4^+^ eosinophils in the peripheral blood of both groups, patients with acute/juvenile form of PCM had more circulating TLR2^+^ eosinophils than controls. There was no difference in TLR4 expression between groups (Fig 5).

Eosinophils can function as antigen-presenting cells and stimulate T cell response *in vivo*. Therefore, they must express cell surface components necessary for antigen presentation and T cell activation (MHC-II, CD80 and CD86) [22, 35]. We next evaluated whether these molecules were differentially expressed in patients and controls.

We demonstrated decreased CD80^+^ circulating eosinophils in PCM patients when compared with the control group. Conversely, we did not find differences in the percentage of CD86^+^ or MHC-II^+^ eosinophils between groups (Fig 5). Altogether, our results suggest that during PCM, peripheral eosinophils are more activated and express more TLR2 than non-infected controls. However, the percentage of CD80^+^ cells is reduced in patients, which could have an inhibitory effect on T cell activation.

These results indicate that during paracoccidioidomycosis there is an activation of eosinophils, revealed by high levels of serum chemokines and granule protein release, increase adhesion and migration capacity and CD69 expression.

### Fungicidal activity of eosinophils against *P*. *brasiliensis* yeast cells

In order to assess differences between PCM patients and healthy controls in eosinophils' ability to kill fungal cells, *P*. *brasiliensis* yeast cells were cultured with eosinophils for 4h and the number of CFU/mL was estimated.

**Fig 6:**
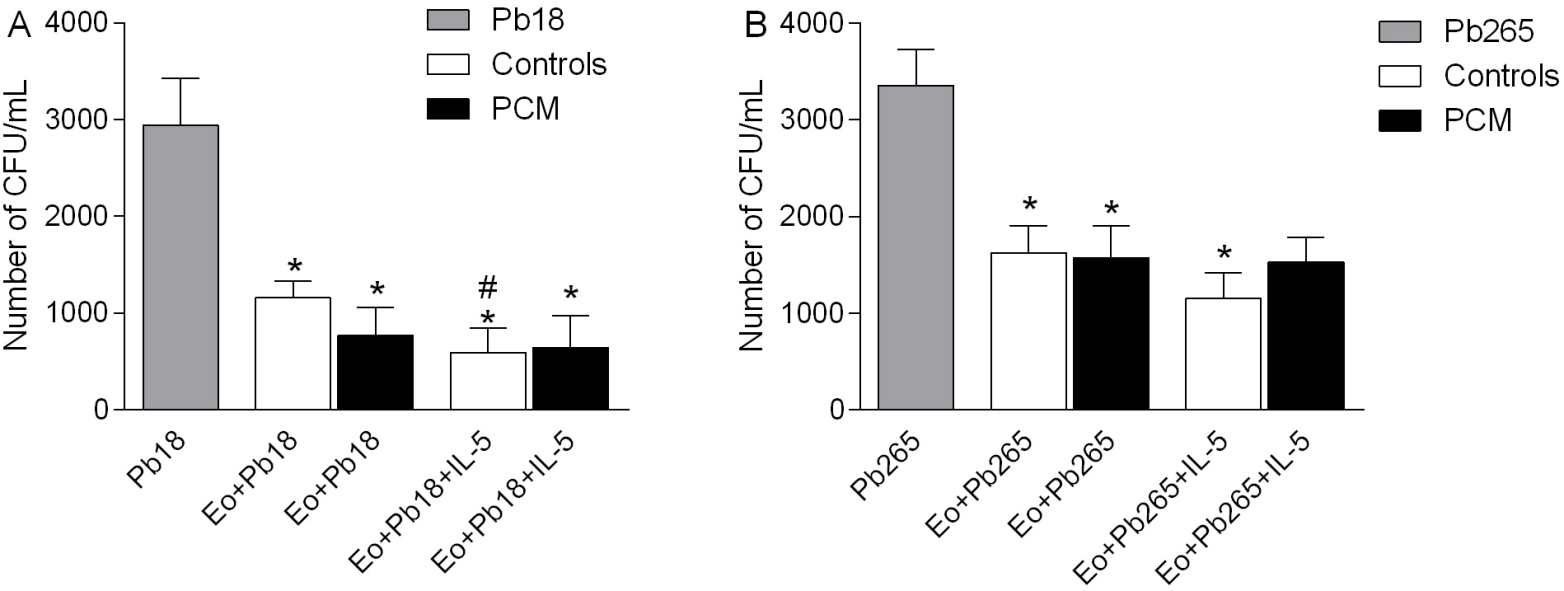
Fungicidal activity of eosinophils against *P*. *brasiliensis* yeasts cells. Number of CFU/mL of *P*. *brasiliensis* strains Pb18 (A) and Pb265 (B) cultured for 4h with eosinophils (Eo) from healthy donors (Controls, n = 8, white bars) or PCM patients (PCM, n = 9, black bars). Alternatively, IL-5 (25ng/mL) was added to the plate wells, as indicated (Eo+Pb18/Pb265+IL-5). Results are expressed as mean ± SEM. *Repeated measures ANOVA*: * p <0.05 compared to Pb18/Pb265 without the addition of eosinophils. # p<0.05 compared to Eo+Pb18.

Eosinophils from healthy donors and patients showed direct cytotoxic activity against *P*. *brasiliensis* yeasts, independent of the virulence of the strain (Fig 6). However, the addition of IL-5 (25ng/mL) induced higher fungicidal activity to Pb18 in controls (p<0.05 in relation to unstimulated eosinophils) than in patients (Fig 6A). In relation to Pb265 eosinophils of PCM patients are also less responsive to IL-5 than controls (Fig 6B).

## Discussion

Peripheral eosinophilia is a common finding in patients with the acute/juvenile form of PCM, especially in children [13, 36, 37]. However, prior studies evaluating the role of these cells in the immune response against *P*. *brasiliensis* were lacking. We analyzed phenotypic and functional characteristics of eosinophils in patients with acute PCM when compared with healthy donors.

We detected high levels of antibodies against *P*.*brasiliensis* and C-reactive protein, a systemic inflammatory marker. Two patients had a negative immunodiffusion test (P10 and P12), despite positive detection of fungal cells in biopsy specimens. ELISA assay detected high titers of antibodies against gp43 of *P*. *brasiliensis* in the serum of both patients. In previous work, we demonstrated that lack of reactivity in immunodiffusion test could be associated with production of low affinity IgG2 class antibodies against carbohydrate epitopes [38]. In accordance with previous reports, all patients presented with mononuclear phagocytic system involvement (lymphadenomegaly and hepasplenomegaly), in addition to bones and skin lesions [39–41].

Activated eosinophils can produce chemokines such as CCL5, CXCL9 and CXCL10. CXCL9 and CXCL10, IFN-γ induced chemokines, are chemotactic for activated T cells and signal through the common receptor CXCR3, which is expressed by memory T cells (CD45RO+) preferably of Th1 and NK cells, but not monocytes or neutrophils [42, 43]. In allergic inflammation, the CC chemokines i.e. CCL5 (CCL5), promote cell migration and activation inflammatory cells, including eosinophils [44]. High concentrations of CCL5, CCL2 (MCP-1), CXCL10 and CXCL9, associated with infiltration of mononuclear cells, were detected in the lungs of *P*. *brasiliensis* infected mice and sera of patients with PCM [45, 46]. In our study we detected high concentrations of CXCL9 and CCL5 in the serum of patients with acute PCM compared to healthy subjects. This may be related to higher eosinophil counts and inflammation typically found in patients with this form PCM. Moreover, biopsies of patients also revealed the presence of MBP, CCL5 and CCL11 in tissues, surrounded by fungal cells. Wagner and colleagues also described eosinophils infiltration and deposit of MBP on *P*. *brasiliensis* yeast cells in granulomas of patients with PCM [47]. These results suggest that eosinophils may assist in the synthesis of chemokines that promote the recruitment of effector T cells and other eosinophils both systemically as well as to the infection site.

We detected higher levels of eosinophil granule proteins in sera of PCM patients when compared to controls. Yang and colleagues have shown that EDN, an eosinophil granule-derived secretory protein, is a TLR2 ligand, and enhances antigen-specific Th2 immune responses [48]. The higher production of EDN by PCM patients may enhance the Th2 polarized immune response present in acute PCM [49–51]. Furthermore, Eosinophils from PCM patients presented a higher migratory capacity in response to CCL11 than eosinophils from controls. However, pre-incubation with both *P*. *brasiliensis* strains did not change this capacity, showing similar results as the unstimulated condition. Eosinophil adhesion to endothelial cells is an important step in the migration process of these cells from blood vessels to the tissues. In this context, CCL11 have a central role by increasing the adhesion and transendothelial migration of eosinophils [17].

We also demonstrated that the adherence of eosinophils to human lung endothelial cells was higher in eosinophils from PCM patients when compared with healthy subjects. The adhered eosinophil numbers were higher in PCM patients, both in basal conditions and when cells were pre-stimulated with CCL11 or IL-5. Our results suggest that eosinophils from acute PCM are more activated, able to produce higher levels of chemokines and granule proteins. Moreover, eosinophils from PCM patients have a higher migratory capacity under CCL11 stimulus and are able to adhere more to lung endothelial cells, which could favor the influx of these cells to inflammatory sites.

Although both migratory and adherence capacity of eosinophils were higher in patients than in controls, no change was observed when the cells were stimulated with fungal cells or chemokines/cytokines (Figs 3 and 4, respectively). These results may imply that eosinophils from patients with the acute form of PCM are already fully activated to migrate and to adhere to endothelial cells.

Accordingly, we next evaluated the expression of CD69, pattern recognition receptors and T-cell activation molecules in eosinophils from PCM patients and healthy controls. As we previously concluded, we found more CD69^+^ eosinophils in the peripheral blood of PCM patients. The same group presents more TLR2^+^ circulating eosinophils. On the other hand, the frequency of CD80^+^ eosinophils in individuals with acute PCM is lower when compared with controls. Besides this apparently increased activation of PCM eosinophils, these patients are not able to clear the infection, and some patients succumb to the disease [39, 52–54].

Loures and colleagues have shown that the presence of TLR2 in mice is associated with susceptibility to *P*. *brasiliensis* infection [55]. In this study using TLR2-deficient mice, the authors reported that *P*. *brasiliensis* might use TLR2 to interact with host cellls, as a fungal virulence mechanism, resulting in lower fungal burden in TLR2^-/-^ mice. The TLR2 deficiency is associated with activation of Th17 cells and lower expansion of regulatory T cells, which leads to an uncontrollled inflammation and tissue damage. A suppressor role of TLR2 was also reported for *Candida albicans*, through induction of IL-10 and Treg [56]. It was also previously demonstrated the presence of TLR2-expressing regulatory DC in the lungs of mice susceptible to *P*. *brasiliensis*, which was not associated with protection [57]. The versatility of recognition by TLR2 is seen as a result from the ability of this receptor to act together with other toll-like receptor, such as TLR1 and TLR6 [58], thus providing proinflammatory (TNF-α and IL-12 production) and anti-inflammatory responses (IL-10 production), a condition which provides an escape strategy used by some pathogens [56, 59]. Indeed, our group has previously reported that PCM patients with active disease present higher numbers of Treg cells, compared to patients who had received treatment or controls [60]. The analysis of Treg cells from PCM patients have shown that these cells presented regulatory phenotype associated with suppressive activity, which may contribute to the onset of the disease. Moreover, the increase in TLR2^+^ population of eosinophils might collaborate to fungal infectiveness, associated with the increase in T regulatory populations, which might lead to a more severe disease, as observed in acute PCM patients. Indeed, it has been suggested that PAMP recognition through TLR by eosinophils is linked to a Th2 phenotype in humans [61].

We next performed a fungicidal assay in order to evaluate the capacity of eosinophils to direct kill *P*. *brasiliensis* yeasts. Our results showed that eosinophils from healthy individuals and PCM patients were able to kill Pb18 and Pb265 yeast cells, although patient cells were less responsive to IL-5 stimulation. These results might indicate that in PCM the fungicidal activity of eosinophils does not account for protection.

Given the importance of the innate immune response leading to protection or immunopathology, and the quality of the adaptive immune response, we believe that eosinophils might have a role in the pathophysiology of the acute form of PCM. In this context, the results of this study confirm the initial hypothesis that eosinophils participate in the early stages of host response to fungi promoting an intense and systemic inflammatory response that result in an inefficient immune response against *P*. *brasiliensis*. Moreover, the higher TLR2 expression suggests that they might have an auxiliary role in the Th2 immune polarization found in patients with acute PCM.
